# Quality modeling of drinking groundwater using GIS in rural communities, northwest of Iran

**DOI:** 10.1186/2052-336X-12-99

**Published:** 2014-06-24

**Authors:** Mohammad Mosaferi, Mojtaba Pourakbar, Mohammad Shakerkhatibi, Esmaeil Fatehifar, Mehdi Belvasi

**Affiliations:** 1 Tabriz Health Services Management Research Center, Tabriz University of Medical Sciences, Tabriz, Iran; 2 School of Health, Student research committee, Tabriz University of medical sciences, Tabriz, Iran; 3 Department of Environmental Health Engineering, Tabriz University of Medical Sciences, Tabriz, Iran; 4 Sahand University of Technology, Sahand, Iran; 5 M.S. Graduated of Geographic Information Systems & Remote Sensing, School of Geography, Tabriz University, Tabriz, Iran

**Keywords:** Heavy metals, GIS, Groundwater, Mapping, Multivariate statistic

## Abstract

Given the importance of groundwater resources in water supply, this work aimed to study quality of drinking groundwater in rural areas in Tabriz county, northwest of Iran. Thirty two groundwater samples from different areas were collected and analyzed in terms of general parameters along with 20 heavy metals (e.g. As, Hg and …). The data of the analyses were applied as an attribute database for preparing thematic maps and showing water quality parameters. Multivariate statistical techniques, including principal component analysis (PCA) and hierarchical cluster analysis (CA) were used to compare and evaluate water quality. The findings showed that hydrochemical faces of the groundwater were of calcium-bicarbonate type. EC values were from 110 to 1750 μs/cm, in which concentration of salts was high in the east and a zone in north of the studied area. Hardness was from 52 to 476 mg/l and CaCO_3_ with average value of 185.88 ± 106.56 mg/L indicated hard water. Dominant cations and anions were Ca^2+^ > Na^+^ > Mg^2+^ > K^+^ and HCO_3_^−^ > Cl^−^ > SO_4_^2−^ > NO_3_^2^, respectively. In the western areas, arsenic contamination was observed as high as 69 μg/L. Moreover, mercury was above the standard level in one of the villages. Eskandar and Olakandi villages had the lowest quality of drinking water. In terms of CA, sampling sites were classified into four clusters of similar water quality and PCA demonstrated that 3 components could cover 84.3% of the parameters. For investigating arsenic anomaly, conducting a comprehensive study in the western part of studied area is strongly recommended.

## Background

Safe drinking water is one of the necessities in sustaining life and a satisfactory (adequate, safe and accessible) supply of water must be available for all people. The advantage of groundwater is that it can be abstracted in many places, which makes pipe transportation unnecessary. Furthermore, water is hygienically reliable and generally it has a constant composition. Sometimes, it can be even distributed without any treatment although a simple and cheap treatment (e.g. disinfection) is often inevitable [1-4].

Due to the rising demand for clean drinking water, management of groundwater quality, especially in developing countries, is very important. It has been reported that approximately one third of the world’s population use groundwater for drinking. Thus, sophisticated monitoring of quality of such resources would play a key role in achieving globally sustainable development in near future [5-7].

After assessing groundwater, it is important to simplify results of the study for policy makers and other stakeholders. So, nowadays, using other sciences such as geographical information system (GIS) has been increased. GIS is a management tool that has grown since the late 20th century. In the past 10 years, the number of GIS users has substantially increased. GIS technology has previously facilitated laborious procedures [8-10]. During the past two decades, various researchers have reported its application in groundwater modeling and quality assessment. Balakrishnan et al. demonstrated spatial variations in groundwater quality using GIS and groundwater quality information maps of the entire studied area in India [11]. Jamshidzadeh and Mirbagheri examined quality and quantity changes in an aquifer in central Iran. Accordingly, using 53 observation wells showed that mean water table declined 0.496 m/year. Also, most of the water samples which were used for quality analysis were not potable [12]. Contamination of groundwater in an area of 180 km^2^ in India was studied by Arumugam and Elangovan; then, they analyzed major anions and cations and found that most of the locations were contaminated by high levels of EC, TDS, K and NO_3_[4]. Another study which was performed by ThiHanh Hoang et al. during 2007–2008 in Mekong River delta in Vietnam found that 26%, 74% and 50% of groundwater samples were above the drinking water guidelines of United State Environmental Protection Agency (USEPA) as far as As (10 mg/L), Mn (0.05 mg/L) and Fe (0.3 mg/L) were concerned, respectively [13].

In the present work, drinking groundwater quality was surveyed and modeled in rural communities of Tabriz in northwest of Iran using GIS. The villages located in Tabriz were of great importance since they had high population compared with other counties in East Azerbaijan province. Uncontrolled expansion of industries, agriculture, settlement and deficiencies in waste management and disposal are considered the threat to surface and groundwater [14]. Moreover, juxtaposition of rural communities with industrial areas around Tabriz county is by itself a threat for the quality of drinking water. There were about 70 villages in Tabriz, only 46 of which were under the coverage of East Azerbaijan Rural Water and Wastewater Company (EARWWC). In these villages, drinking water was mainly provided from groundwater (spring, well or qanat) and water distribution system of Tabriz county was used in some parts. The objective of present study was to evaluate drinking groundwater quality using different method such as mapping of quality parameters along with heavy metals of concern, multivariate statistical analysis to classify water resources and determining hydrochemical faces of the groundwater.

## Methods

### Studied area

**Figure 1:**
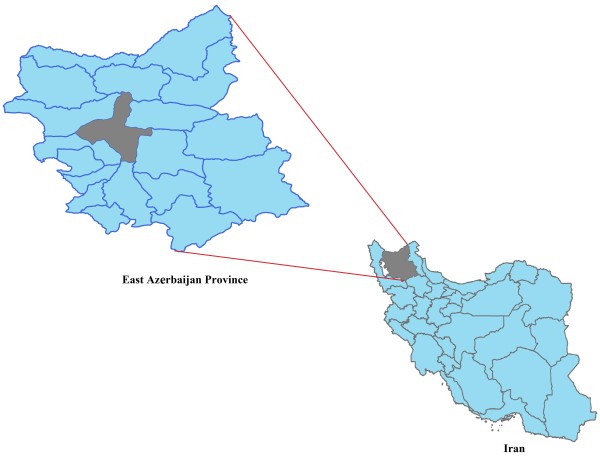
Location of the studied area (Tabriz) on Iran map.

Tabriz is located in central part of East Azerbaijan province with the area of about 2167.2 km^2^ which is 4.76% of total area of the province (Figure 1). It is subdivided into two districts: central district and Khosrowshahr district with five cities: Tabriz, Basmenj, Sardrud, MalekKian and Khosrowshahr. Tabriz, as the center of this province, is the fifth largest city and one of the historical capitals of Iran which is situated at the altitude of 1,350 m above sea level.

### Water sampling and analysis

Drinking water of the villages around Tabriz was provided from either groundwater sources (wells, springs and qanats) or urban distribution system. Only the villages using groundwater sources which were under the coverage of EARWWC were included in this study.

In order to assess groundwater quality, sampling was done in 2012 and 32 samples were totally gathered during the study. The samples were collected in acid-washed PET bottles after 5 min discharge of the current and 3 times washing of the bottles. Parameters including pH, electric conductivity (EC), hardness, major cations (Ca^2+^, Mg^2+^, Na^+^ and K^+^) and major anions (HCO_3_^−^, SO_4_^2−^, Cl^−^, F^−^ and NO_3_^2−^) were considered to be measured. All the analyses were according to standard methods for examination of water and wastewater [15]. within addition to the above mentioned parameters, heavy metals such as arsenic, aluminum, boron, barium, beryllium, chromium, copper, mercury, tin, zink, cadmium, cobalt, iron, manganese, molybdenum, nickel, lead, antimony, selenium and vanadium were analyzed by ICPOES (inductively coupled plasma optical emission spectrometry) method. Due to some financial limitations, only 18 out of 32 samples were analyzed in terms of the presence of heavy metals. In order to applicability of the research, samples for heavy metal analysis were selected according to the recommendations made by experts of the EARWWC considering previous available analysis results made by EARWWC. Also, population of village was considered as another important criteria for selected villages.

All the data were entered into a spatial database and spatial variations of the results were developed using inverse distance weighting (IDW) method. Arc GIS software (version 10.0) was also applied for developing maps. IDW interpolation assumes that each measured point has a local influence that diminishes with distance. Thus, points in the near neighborhood are given high weights, whereas points at a far distance are given small weights. The general formula of IDW interpolation for 2-D problems is the following:

$$\begin{array}{cc} w\left(x,y\right)={\sum_{i=1}^{N}}_{i}w_{i} & {}_{i}=\frac{{\left(\frac{1}{d_{i}}\right)}^{p}}{\sum_{i=1}^{N}{\left(\frac{1}{d_{i}}\right)}^{p}} \end{array}$$

Where *w(x,y)* is the predicted value at location *(x,y)*, *N* is the number of nearest known points surrounding *(x,y)*, *ƛ*_*i*_ are the weights assigned to each known point value w_i_ at location *(x*_*i*_*,y*_*i*_*)*, *d*_*i*_ are the 2-D Euclidean distances between each *(x*_*i*_*,y*_*i*_*)* and *(x,y)*, and p is the experiment which influences the weighting of *w*_*i*_ on *w*[16]. The advantage of IDW is that it is intuitive and efficient, that’s why IDW method is widely used in spatial interpolation of groundwater quality [11].

In order to clarify results of this work, piper diagrams were plotted using AqQa software and then interpreted.

### Multivariate statistical analysis

Descriptive statistics (including means, maximum, minimum and standard deviations), logistic regression model, correlation and analysis of variance (ANOVA) tests were done to analyze water quality data and their relationships. Also, principal component analysis (PCA) method (rotation method Varimax, Kaiser Normalization) and cluster analysis (CA) were used to group the related water quality parameters. Varimax rotation is the most widely used orthogonal rotation in PCA for easier interpretation of results [17-19]. Hierarchical agglomerative clustering is the most common approach which provides instinctive similarity relationships between any one sample and the entire dataset and is typically illustrated by a dendrogram (tree diagram) [20].

## Results and discussion

### Physicochemical analysis

Results of the hydrochemical analysis along with descriptive statistics of quality parameters of the groundwater samples which were taken from the studied area are presented in Table 1.

**Table 1:** Hydrochemical analysis of the groundwater samples taken from the study area

**No**	**Villages**	**Cations (mg/L)**				**Anions (mg/L)**					**pH**	**EC (μs/cm)**	**TDS (mg/L)**	**Hardness (mg/L)**
		**Ca**	**Mg**	**Na**	**K**	**F**	**Cl**	**SO**_**4**_	**HCO**_**3**_	**NO**_**3**_				
1	Hervi	40.0	19.4	6.1	5.0	0.20	26	16.2	175.7	0.4	6.76	367	238.6	180
2	Eskandar	160.0	18.5	160.0	9.0	0.20	190	164.6	414.8	59.4	6.67	1750	1137.5	476
3	Ligvan	32.0	16.5	3.6	3.0	0.60	20	1.8	146.4	3.9	6.88	294	191.1	148
4	Shadabad	24.0	29.2	29.5	5.0	0.20	30	45.5	180.6	4.0	6.93	470	305.5	180
5	Esparakhun	8.0	14.6	4.0	1.0	0.17	12	2.2	78.1	2.1	7.10	165	107.3	80
6	Kondrud	80.0	30.1	51.6	6.5	0.20	72	74.6	297.7	9.4	6.48	837	544.1	324
7	Dizaj leylikhani	24.0	14.6	11.0	4.0	0.15	10	12.2	126.9	7.4	7.13	289	187.9	120
8	Asbes	40.0	29.2	86.1	5.0	0.40	94	77.6	244.0	13.5	6.81	881	572.7	220
9	Asenjan	88.0	19.4	71.4	6.5	0.20	124	107.6	219.6	15.0	7.20	926	601.9	300
10	Varanag	56.0	21.4	61.5	7.1	0.20	54	60.4	258.6	10.5	6.80	664	431.6	228
11	Ola kandi	44.8	70.0	87.4	16.0	0.40	30	220.5	439.2	5.4	6.89	1232	800.8	400
12	Esfahlan	40.0	63.2	73.8	9.9	0.20	32	195.6	302.6	15.0	6.46	1024	665.6	360
13	Anarjan	40.0	34.0	22.1	4.8	0.20	36	46.4	239.1	5.2	6.09	552	358.8	240
14	Komanj olya	16.0	14.6	4.5	0.9	0.20	4	5.4	92.7	6.5	6.34	205	133.3	100
15	Zinjanab	24.0	4.9	4.5	2.0	0.20	12	9.9	53.7	5.0	7.49	168	109.2	80
16	Espiran1	40.0	26.2	137.8	9.0	0.40	110	173.9	175.7	16.2	7.41	1010	656.5	208
17	Espiran2	43.2	17.5	115.6	9.0	0.30	100	179.5	146.4	11.4	7.42	930	604.5	180
18	Khellejan	48.0	19.4	46.7	8.0	0.30	60	49.6	209.8	9.3	6.54	685	445.3	200
19	Shadabad olya	27.1	6.6	27.7	3.4	0.22	25	20.0	126.9	7.1	7.66	303	197.0	96
20	Zaranag	24.0	5.7	21.0	3.2	0.23	11	12.0	122.0	6.2	7.74	245	159.3	84
21	Bagh yagub	41.5	15.2	41.5	4.1	0.52	25	34.0	229.4	8.4	7.55	487	316.6	168
22	Nemat abad	27.1	7.6	29.6	3.5	0.23	25	22.0	126.9	9.7	7.52	310	201.5	100
23	Fath abad	27.1	9.5	27.9	3.4	0.18	25	24.0	122.0	13.3	7.64	315	204.8	108
24	Hezar baran	62.3	19.0	52.5	7.3	0.49	57	55.0	258.6	12.8	7.50	667	433.6	236
25	Beirag	35.1	7.6	22.8	4.4	0.34	11	13.0	165.9	9.3	7.63	318	206.7	120
26	Jangur	22.4	7.6	24.1	3.5	0.54	14	13.0	122.0	11.1	7.71	273	177.5	88
27	Chavan	19.2	8.5	16.9	3.7	0.18	7	8.0	117.1	13.3	8.02	230	149.5	84
28	Gollujeh	12.8	4.7	3.9	0.1	0.90	4	3.0	39.0	22.1	7.12	110	71.5	52
29	Nosrat abad	17.6	3.8	5.4	0.4	0.14	4	4.0	58.6	17.7	7.52	131	85.2	60
30	Anakhatun	44.7	15.2	22.4	3.1	0.27	32	32.0	170.8	15.9	7.76	422	274.3	176
31	Karjan	63.9	12.3	57.5	6.2	0.41	68	70.0	190.3	28.8	7.51	678	440.7	212
32	Aghaj oghli	61.2	44.3	91.3	4.3	0.52	107	72.3	372.5	6.1	7.64	988	642.2	340
	Maximum	160.0	70.0	160.0	16.0	0.90	190	220.5	439.2	59.4	8.02	1750	1137.5	476
	Minimum	8.0	3.8	3.6	0.1	0.14	4	1.8	39.0	0.4	6.09	110	71.5	52
	Mean	41.7	19.7	44.4	5.1	0.31	44.6	57.1	188.2	11.9	7.19	560	364.1	185.9
	Std. deviation	28.57	15.54	40.76	3.26	0.17	43.6	63.17	99.14	10.4	0.49	382.90	248.9	106.56

**Figure 2:**
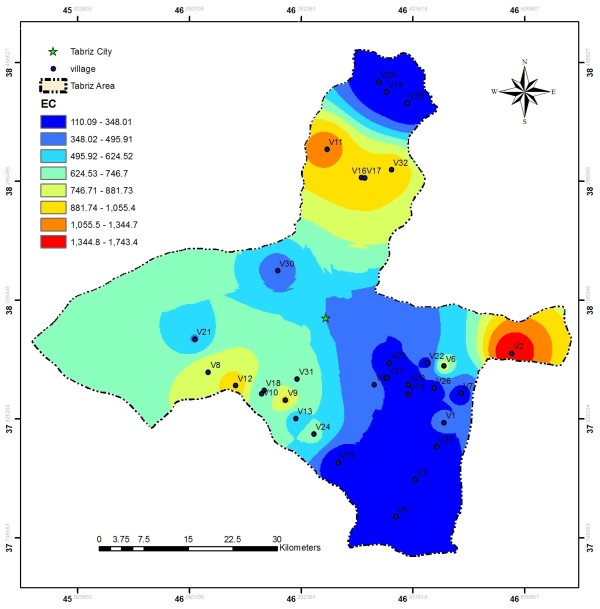
EC variation in the studied area.

**Figure 3:**
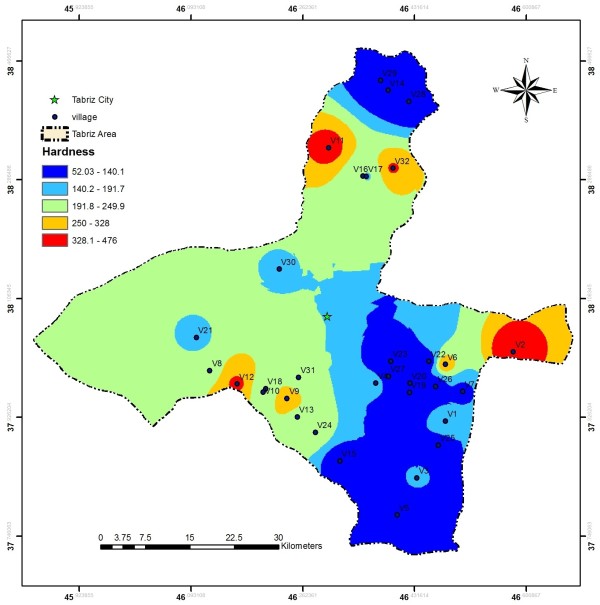
Hardness distribution in the considered area.

**Figure 4:**
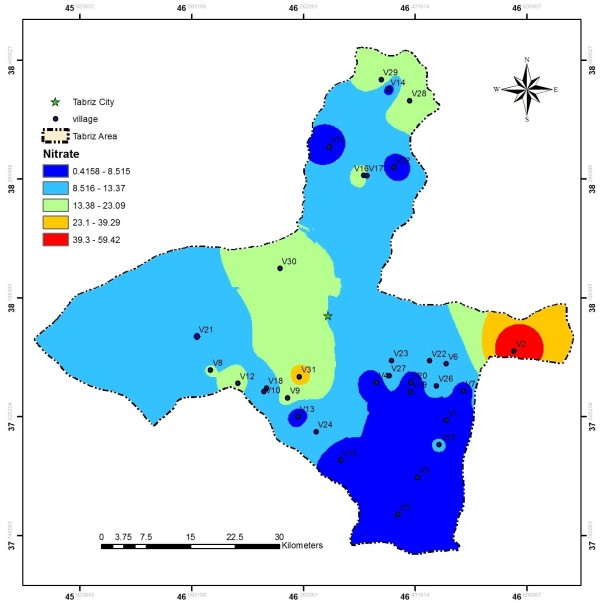
Nitrate distribution in the studied area.

**Figure 5:**
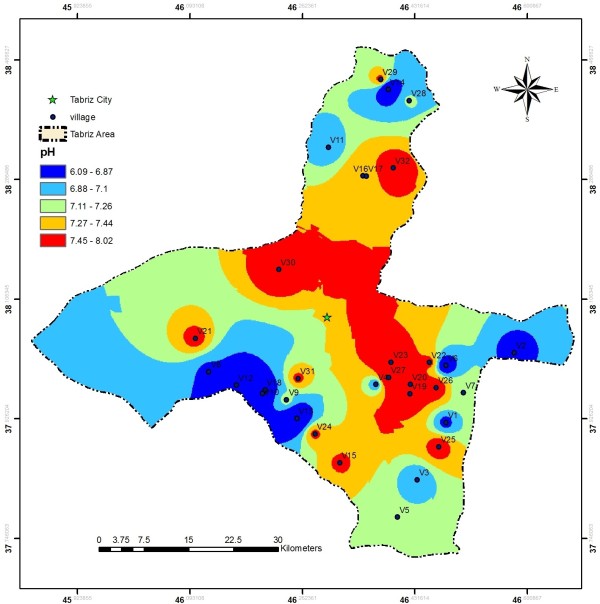
pH distribution in the studied area.

**Figure 6:**
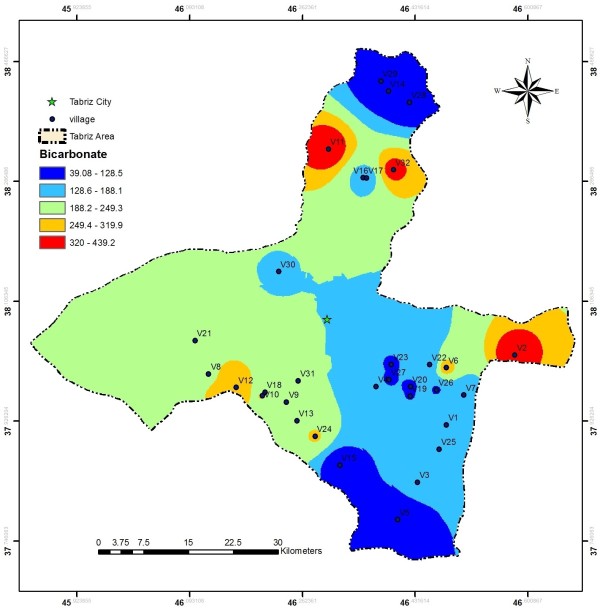
Bicarbonate distribution in the studied area.

**Figure 7:**
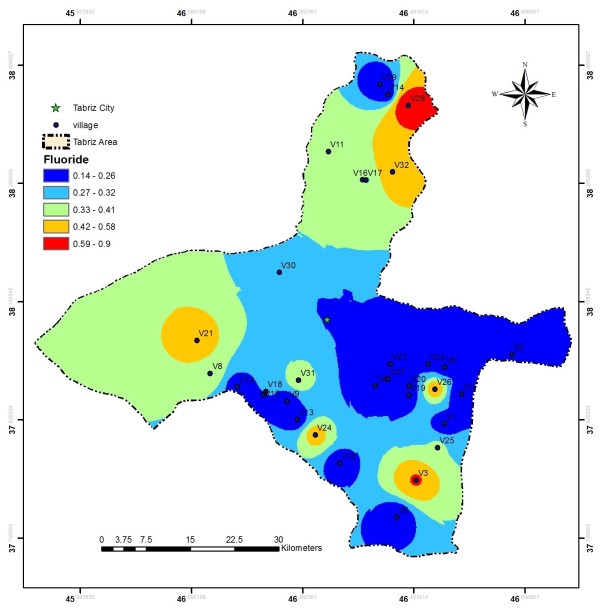
Fluoride distribution in the studied area.

EC values in the investigated area ranged from 110 to 1750 μs/cm, these values are much less than the values reported by Baghvand et al. in an aquifer in Iran central desert (1987–12751 μs/cm) [7]. Figure 2 represents EC variation in Tabriz, indicating high concentration of salts in east and a zone in north of the studied area. In terms of hardness, water is grouped as soft water (>75 mg/L CaCO_3_), medium hard (75–150 mg/L CaCO_3_), hard water (150–300 mg/L CaCO_3_) and very hard water (>300 mg/L CaCO_3_). Range and average values of total hardness in the sampled water were 52 to 476 and 185.88 ± 106.56 mg/L as CaCO_3_, respectively, representing that the studied water samples could be grouped as hard water (Figure 3). In a similar study in India, majority of the samples fall in very hard water category (>300 mg/L CaCO_3_) [11]. Nitrate values, as an important parameter regarding its health effects, varied from 0.4 to 59.4 mg/L with average value of 11.91 ± 10.49. In the similar study in India, Nitrate concentration in 73.68% of samples exceeded the guideline value (50 mg/L) [11]. Nitrate distribution is given in Figure 4, according to which only, in village 2 (Eskandar), level of nitrate exceeded guideline value (50 mg/L). Figures 5, 6, 7 show pH, bicarbonate and fluoride variations in the studied area, respectively. As can be seen, in most parts of the studied area, pH values were above 7; however, in some parts like north, east and southwest, this value was below 7. In many parts of the considered area, bicarbonate concentration was below 250 mg/L as CaCO_3_. For fluoride, concentration was less than 0.5 mg/L, except in villages 3 and 28.

### Hydrochemical faces

**Figure 8:**
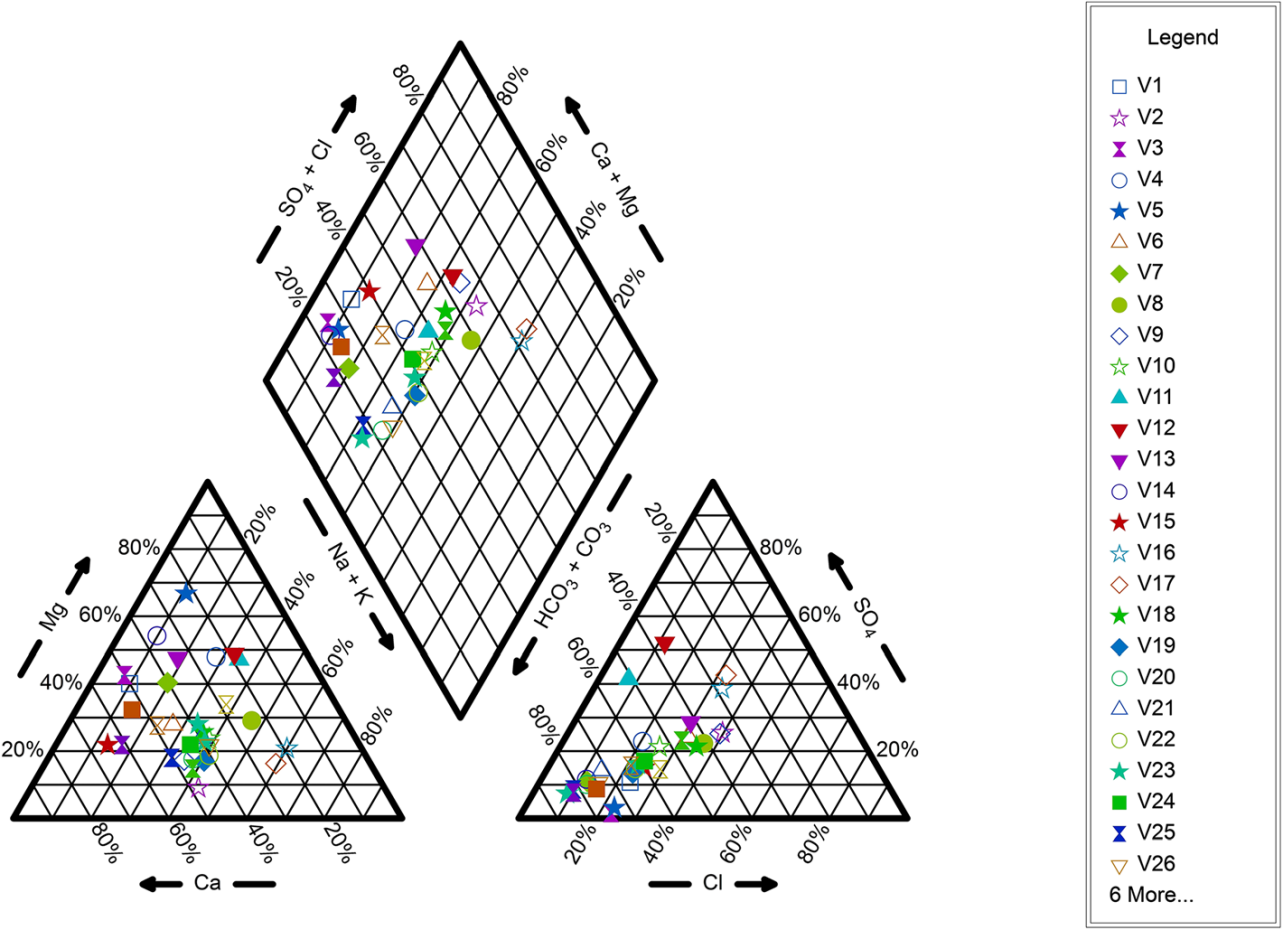
Piper diagram of the sampling points.

Tables 2 and 3 demonstrate percentage of constituents at the sampled points and hydrochemical faces of the groundwater according to piper diagram, respectively. According to these tables, in most parts of the considered area, hydrochemical faces of the groundwater were of calcium-bicarbonate type. Piper diagram of the studied area is given in Figure 8. According to the diagram, groundwater was divided to six faces [4,12,21]. In the present investigation, bicarbonate was the most dominant anion (in 93% of the samples) and calcium was the most dominant cation (in 65% of the samples). Therefore, chemical characteristic of the water was dominated by Ca HCO_3_ type water.

**Table 2:** Percentage of constituents of the groundwater samples and ion balance

**Villages**	**Cations (%)**				**Anions (%)**					**Ion balance**		
	**Ca**	**Mg**	**Na**	**K**	**F**	**Cl**	**SO**_**4**_	**HCO**_**3**_	**NO**_**3**_	**Σ Cations (meq/l)**	**Σ Anions (meq/l)**	**Error**
V1	50.06	40.13	6.61	3.21	0.27	18.53	8.53	72.51	0.17	3.99	3.96	−0.36
V2	47.83	9.11	41.68	1.38	0.06	32.41	20.73	40.99	5.80	16.69	16.53	−0.47
V3	50.06	42.64	4.90	2.41	1.02	18.27	1.24	77.45	2.02	3.19	3.09	−1.61
V4	23.92	47.93	25.60	2.55	0.22	17.56	19.67	61.22	1.33	5.01	4.82	−1.91
V5	22.19	66.71	9.67	1.42	0.53	19.88	2.68	74.92	2.00	1.80	1.70	0.10
V6	44.93	27.91	25.28	1.87	0.12	23.59	18.05	56.49	1.75	8.88	8.61	−1.57
V7	40.22	40.30	16.05	3.44	0.29	10.31	9.28	75.77	4.36	2.98	2.74	0.24
V8	24.13	29.02	45.30	1.55	0.25	31.22	19.03	46.94	2.56	8.27	8.49	1.33
V9	47.42	17.28	33.51	1.80	0.11	36.51	23.39	37.46	2.53	9.26	9.58	1.69
V10	37.71	23.75	36.09	2.45	0.15	21.19	17.51	58.80	2.35	7.41	7.19	−1.53
V11	18.32	47.19	31.14	3.35	0.17	6.65	36.09	56.41	0.68	12.21	12.72	2.07
V12	18.72	48.78	30.12	2.38	0.10	8.87	40.04	48.61	2.37	10.66	10.17	−2.35
V13	33.95	47.63	16.33	2.09	0.18	16.97	16.15	65.31	1.40	5.88	5.98	0.88
V14	36.01	54.12	8.83	1.04	0.57	6.08	6.00	81.66	5.69	2.22	1.86	0.36
V15	64.93	21.69	10.61	2.77	0.70	22.38	13.64	57.98	5.31	1.84	1.51	0.33
V16	19.23	20.81	57.74	2.22	0.21	31.42	36.65	29.06	2.65	10.38	9.88	−2.48
V17	24.35	16.26	56.79	2.60	0.17	30.83	40.84	26.15	2.00	8.85	9.15	1.64
V18	38.44	25.68	32.61	3.28	0.25	26.79	16.33	54.27	2.36	6.23	6.32	0.69
V19	42.39	17.12	37.76	2.73	0.35	21.16	12.55	62.50	3.44	3.20	3.32	1.87
V20	44.94	17.63	34.36	3.07	0.46	11.33	9.40	75.05	3.76	2.66	2.66	−0.07
V21	39.61	23.91	34.49	1.98	0.51	13.19	13.30	70.44	2.55	5.23	5.32	0.86
V22	40.34	18.62	38.36	2.68	0.36	20.63	13.45	60.94	4.62	3.36	3.40	0.65
V23	39.42	22.75	35.30	2.54	0.28	20.53	14.61	58.31	6.27	3.44	3.42	−0.26
V24	43.51	21.89	31.99	2.61	0.36	22.26	15.88	58.63	2.87	7.14	7.21	0.46
V25	50.32	17.95	28.51	3.21	0.52	8.72	7.84	78.58	4.35	3.48	3.45	−0.48
V26	38.73	21.71	36.44	3.12	0.99	13.97	9.42	69.41	6.22	2.88	2.87	−0.14
V27	38.39	28.24	29.54	3.83	0.38	8.01	6.65	76.41	8.55	2.49	2.50	0.27
V28	53.09	32.55	14.21	0.15	3.93	8.32	5.18	52.94	29.63	1.20	1.21	0.18
V29	61.08	21.79	16.43	0.70	0.51	7.00	5.81	66.75	19.93	1.44	1.43	−0.06
V30	49.19	27.58	21.49	1.74	0.31	19.49	14.38	60.27	5.55	4.54	4.63	1.04
V31	46.43	14.80	36.45	2.31	0.31	27.39	20.93	44.70	6.67	6.86	6.96	0.67
V32	50.06	40.13	6.61	3.21	0.26	18.53	8.53	72.51	0.17	10.78	10.73	−0.28

**Table 3:** Hydrochemical faces of groundwater

**Faces**	**Sample ID**	**Number of samples**	**Percentage of samples**
CaHCO_3_	V1, V3, V4, V5, V7, V10, V11,V14, V15, V18, V19, V20, V21, V22, V24, V25, V26,V 27, V28, V29, V30, V32	22	68.75
NaCl	V16, V17	2	6.25
Mixed CaNa HCO_3_	Nill	Nill	Nill
Mixed CaMgCl	V2, V6, V8, V9, V12, V13, V23, V31	8	25
CaCl	Nill	Nill	Nill
NaHCO_3_	Nill	Nill	Nill

### Heavy metals

Table 4 shows concentration of heavy metals and trace elements in the groundwater samples. In addition to the heavy metals presented in Table 4, some other elements including cadmium, cobalt, iron, manganese, molybdenum, nickel, lead, antimony, selenium and vanadium, were either analyzed which their concentrations were non-detectable in samples.

**Table 4:** Concentration of heavy metals in groundwater samples (in μg/L)

**No**	**Villages**	**As**	**Al**	**B**	**Ba**	**Be**	**Cr**	**Cu**	**Hg**	**Sn**	**Zn**
V1	Hervi	> 1	1.89	450.16	56	4.85	23.28	29.00	> 1	> 9	> 2
V2	Eskandar	> 1	0.78	2074.76	105	4.83	22.26	31.72	6.50	19.57	2.87
V3	Ligvan	> 1	18.04	161.80	54	5.37	21.42	36.25	> 1	> 9	> 2
V4	Shadabad	22.43	15.81	500.82	34	5.61	20.35	37.68	> 1	> 9	> 2
V5	Esparakhun	> 1	147.11	61.84	8	5.54	21.48	42.30	> 1	> 9	> 2
V6	Kondrud	19.93	3.69	477.01	36	4.65	23.58	29.55	> 1	> 9	> 2
V7	Dizaj leylikhani	> 1	2.94	112.19	49	5.13	23.52	49.82	> 1	> 9	26.46
V8	Asbes	69.15	12.25	890.65	22	5.63	21.50	35.91	> 1	> 9	4.56
V9	Asenjan	> 1	4.31	504.23	99	4.84	23.42	30.55	> 1	> 9	131.10
V10	Varanag	24.19	4.13	519.78	52	5.10	23.21	28.83	> 1	10.80	25.26
V11	Ola kandi	> 1	2.36	1510.82	50	4.90	22.26	29.89	> 1	19.25	5.56
V12	Esfahlan	42.57	5.34	853.97	25	5.25	21.86	32.48	> 1	12.04	15.96
V13	Anarjan	> 1	7.76	277.97	69	4.86	23.46	29.47	> 1	> 9	10.54
V14	Komanj olya	> 1	101.39	45.01	14	5.33	22.49	32.91	> 1	> 9	> 2
V15	Zinjanab	> 1	19.74	51.45	5	5.29	22.26	36.72	> 1	> 9	5.59
V16	Espiran1	> 1	14.26	708.16	40	4.76	23.84	29.62	> 1	9.38	> 2
V17	Espiran2	> 1	2.27	1066.07	27	5.03	23.03	33.23	> 1	11.97	8.27
V18	Khellejan	36.07	3.04	734.88	47	5.19	22.86	33.70	> 1	10.57	> 2
-	Standard values	10	100	2400	700	12	50	2000	6	-	-

**Figure 9:**
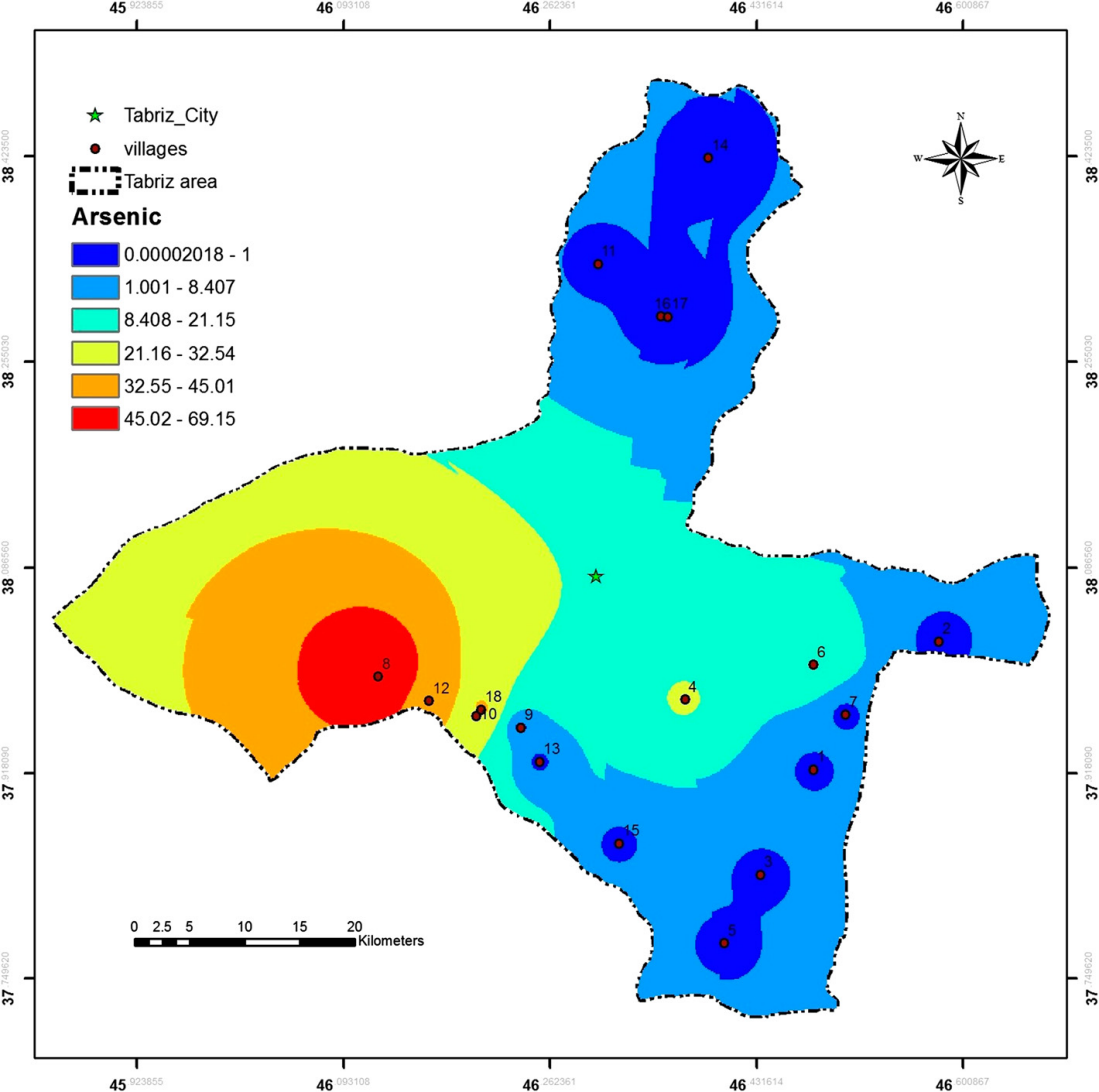
Arsenic distribution in the studied area.

One of the heavy metals of concern in the studied area was arsenic, which is a naturally occurring contaminant in groundwater. Exposure to high levels of arsenic can cause short-term or acute symptoms as well as long-term or chronic health effects. Arsenic in groundwater is found largely due to the minerals associated with previous volcanic activities dissolving from weathered rocks, ash and soils [22-24]. However, arsenic concentrations of greater than 10 μg/L (guideline value of World Health Organization for arsenic in drinking water [23]) were detected in the water supplies, especially in western district of the considered area (Figure 9). In Shadabad, Kondrud, Asbes, Varanag, Esfahlan and Khellejan villages, concentration of above 10 μg/L was found.

The presence of aluminum at concentrations exceeding 0.1–0.2 mg/l often leads to consumer complaints due to its deposition of aluminum hydroxide floc. There is no health-based guideline value for the concentration of aluminum in drinking water; however, aluminum concentrations of less than 0.1 mg/L are achievable in many circumstances [23]. Results of this investigation demonstrated that only 2 of the samples had aluminum concentrations of more than 0.1 mg/L.

Inorganic mercury is a predominate form that is found in surface and groundwater, usually at concentrations of below 0.5 μg/L; however, local mineral deposits may produce higher levels in groundwater [23]. As depicted in Table 4, only in one case, mercury concentration was above the guideline value (6 μg/L). Main problems caused by inorganic mercuric poisoning include liver and renal damage which lead to death [25]. Organicmethylmercury affects the central nervous system. Relatively few instances of elevated concentrations of mercury in groundwater have been reported, except when mercury is included among other contaminants in site-specific hazardous waste investigations or other studies of groundwater contamination in industrialized areas [26,27].

Other heavy metal values presented in Table 4 were below the standard values.

### Statistical analysis

Table 5 provides a matrix of correlation coefficients between quality parameters of the analyzed water samples along with heavy metals. Values of high correlation are specified in bold. As can be observed in the table, there was high correlation between total hardness, Ca^2+^, HCO_3_^−^, Mg^2+,^ K^+^, Na^+^, SO_4_^2−^, Cl^−^, B and Sn. Furthermore, there was significant correlation between Na^+^, SO_4_^2−^, NO_3_^2−^ and B. The correlation between Ca^2+^, NO_3_^2−^ and Hg was also significant. As can be observed in Table 5, the correlation between Cr and Br was negatively significant.

**Table 5:** Matrix of correlation coefficients between the analyzed parameters

	**EC**	**Hardness**	**pH**	**Ca**^**2+**^	**Mg**^**2+**^	**Na**^**+**^	**K**^**+**^	**HCO**_**3**_^**−**^	**SO**_**4**_^**2−**^	**Cl**^**−**^	**NO**_**3**_^**2−**^	**F**^**−**^	**Al**	**As**	**Hg**	**B**	**Ba**	**Br**	**Cr**	**Cu**	**Sn**	**Zn**
EC	1																					
Hardness	**0.94**	1																				
pH	−0.33	−0.45	1																			
Ca^2+^	**0.82**	**0.81**	−0.25	1																		
Mg^2+^	0.65	**0.76**	−0.49	0.24	1																	
Na^+^	**0.93**	**0.76**	−0.12	**0.71**	0.47	1																
K^+^	**0.82**	**0.78**	−0.281	0.51	**0.73**	**0.73**	1															
HCO_3_^−^	**0.87**	**0.95**	−0.36	**0.72**	**0.78**	0.67	**0.78**	1														
SO_4_^2−^	**0.89**	**0.79**	−0.27	0.53	**0.72**	**0.87**	**0.89**	**0.70**	1													
Cl^−^	**0.86**	**0.73**	−0.18	**0.86**	0.26	**0.89**	0.51	0.62	0.65	1												
NO_3_^2−^	0.53	0.41	−0.01	**0.71**	−0.10	0.55	0.20	0.29	0.35	0.61	1											
F^−^	−0.01	−0.04	0.15	−0.07	0.01	0.05	−0.05	0.03	−0.02	−0.01	0.07	1										
Al	−0.49	−0.51	−0.01	−0.44	−0.28	−0.40	−0.57	−0.50	−0.41	−0.38	−0.24	−0.18	1									
As	0.17	0.15	−0.29	−0.04	0.29	0.14	0.11	0.23	0.09	0.08	0.01	0.11	−0.20	1								
Hg	0.62	0.57	−0.12	**0.82**	−0.11	0.54	0.19	0.48	0.28	0.67	**0.93**	−0.13	−0.13	−0.15	1							
B	**0.93**	**0.82**	−0.04	**0.71**	0.45	**0.85**	**0.79**	**0.82**	**0.80**	**0.72**	**0.75**	0.16	−0.43	0.15	0.68	1						
Ba	0.53	0.61	−0.17	**0.76**	0.03	0.37	0.34	0.54	0.24	0.60	0.56	−0.01	−0.50	−0.26	0.55	0.45	1					
Br	−0.48	−0.53	0.07	−0.56	−0.15	−0.41	−0.49	−0.45	−0.43	−0.44	−0.3	0.12	0.47	0.44	−0.24	−0.33	−0.58	1				
Cr	0.13	0.01	0.00	0.23	−0.13	0.18	0.18	0.05	0.12	0.23	0.04	−0.22	−0.32	−0.32	−0.08	−0.05	0.33	**−0.81**	1			
Cu	−0.48	−0.53	0.33	−0.42	−0.34	−0.41	−0.47	−0.50	−0.45	−0.38	−0.12	−0.13	0.33	−0.02	−0.01	−0.38	−0.32	0.57	−0.33	1		
Sn	**0.80**	**0.74**	−0.03	0.54	0.52	**0.72**	**0.83**	**0.75**	**0.79**	0.46	0.62	0.14	−0.30	−0.03	0.58	**0.89**	0.34	−0.34	0.00	−0.33	1	
Zn	0.12	0.17	0.22	0.27	−0.06	0.08	0.05	0.04	0.10	0.29	0.07	−0.21	−0.17	−0.12	−0.09	−0.07	0.51	−0.23	0.29	−0.08	−0.14	1

Table 6 demonstrates rotated factor loadings for water quality parameters. In KMO and Bartlett’s Test, p > 0.001 with coefficient of 0.658 was significant. PCA showed that 3 components could cover 84.3% of the parameters. In 32 analyzed water samples, according to rotated component matrix with 3 factor solution, PC1 accounted for more than 60.3% of total variance in the dataset and was loaded with magnesium, potassium, bicarbonate, sulfate, hardness and electric conductivity; i.e. these parameters demonstrated a similar behavior in groundwater. The second component (PC2) explaining 14.8% of total variance had strong positive loadings for nitrate, calcium, chloride and sodium. The third component (PC3) of PCA demonstrated that only 9.1% of total variation had positive loading of pH and fluoride.

**Table 6:** Rotated factor loadings of PCA application for water quality parameters

**Parameters**	**PC1**	**PC2**	**PC3**
**Mg**^**2+**^	**0.959**	−0.093	−0.096
**K**^**+**^	**0.853**	0.289	−0.008
**HCO**_**3**_^**−**^	**0.820**	0.411	−0.074
**SO**_**4**_^**2−**^	**0.806**	0.425	0.041
**Hardness**	**0.796**	0.526	−0.166
**EC**	**0.734**	0.673	−0.040
**NO**_**3**_^**2−**^	−0.062	**0.884**	0.062
**Ca**^**2+**^	0.330	**0.867**	−0.168
**Cl**^**−**^	0.371	**0.856**	−0.017
**Na**^**+**^	0.587	**0.722**	0.132
**F**^**−**^	0.100	−0.049	**0.868**
**pH**	−0.477	0.049	**0.575**
**Eigenvalue**	7.244	1.780	1.092
**Variance %**	60.366	14.833	9.102
**Cumulative %**	60.366	75.199	84.301

Numbers in bold indicate effective parameter in each PC.

**Figure 10:**
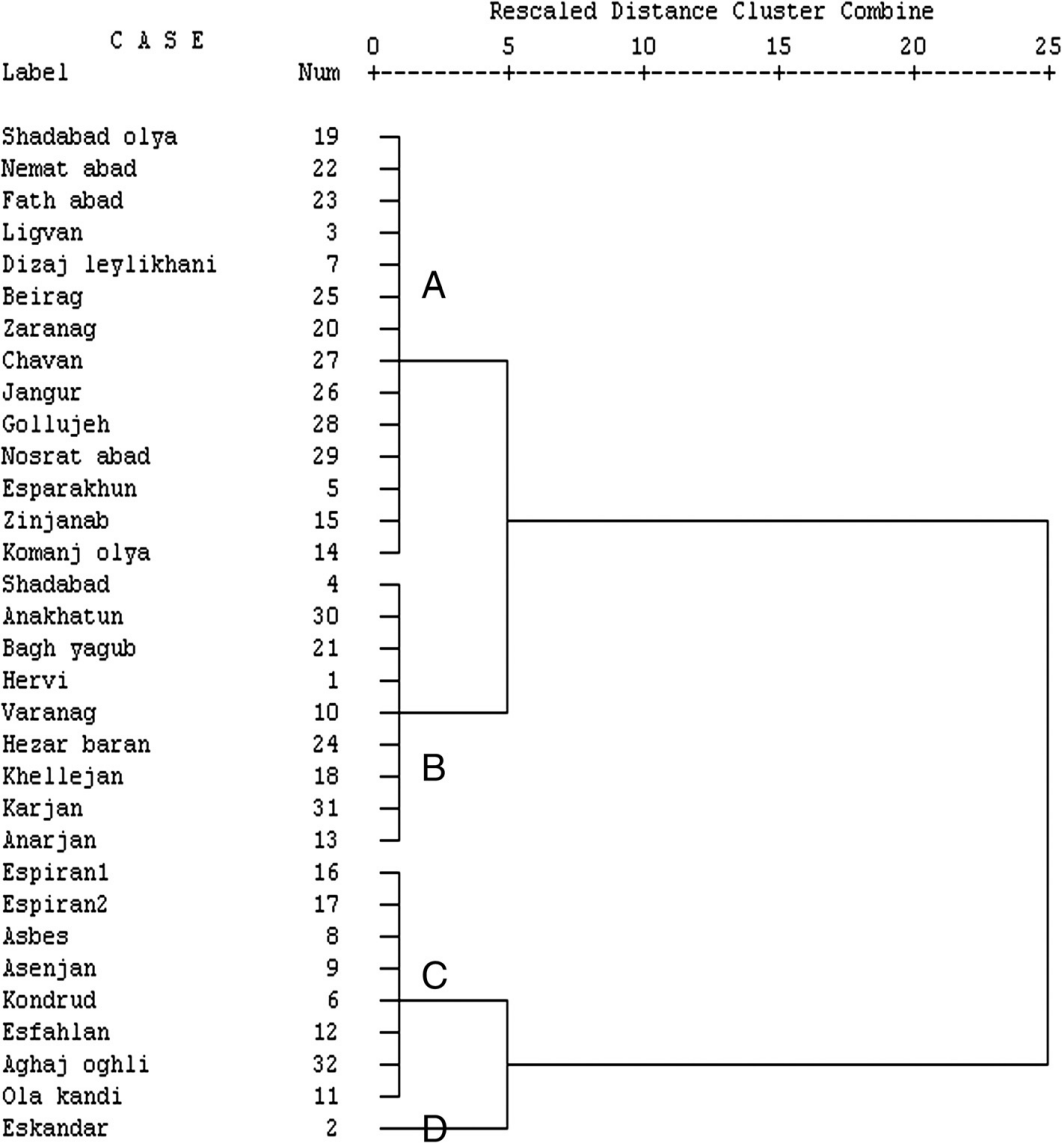
Dendrogram showing cluster analysis of quality parameters.

In KMO and Bartlett’s test for heavy metals, p > 0.001 with coefficient of 0.535 was considered significant. Principal component analysis showed that three components explained 72.39 percent of variance (Table 7). Using rotated component matrix with 3 factor solution, PC1 included boron, tin and mercury. PC2 contained copper, zinc, barium, chromium and beryllium and PC3 was loaded with arsenic and aluminum. In PC2, zinc, barium and chromium had reverse correlation with beryllium and copper and also there was reverse correlation between arsenic and aluminum in PC3.Figure 10 demonstrated the dendrogram obtained by CA for water quality parameters. The figure indicates relationship and similarity between water resources. This dendrogram introduced four distinct groups as A, B, C and D. Considering the location of different villages in the already presented quality maps, it can be observed that the villages grouped in cluster A (3, 5, 7,14, 15,19, 20, 22, 23, 25, 26, 27, 28 and 29) were mainly located in southern and northern parts of the studied area, the ones grouped in cluster B (1, 4, 10, 13, 18, 21, 24, 30 and 31) were distributed in western part of this area and those located in cluster C (6, 8, 9, 11, 12, 16, 17 and 32) were seen in different parts. Finally, the dendrogram clarified abnormality of the water sample from Eskandar village which constituted one group as cluster. As shown in Figure 10 and the results presented in tables and maps thus far, quality of water in this village was infelicitous compared to other villages, which could be due to the fact that water well of this village was located close to the river and in the agricultural area. Since water table level in this area had high intrusion of contaminants through river, which passed through the village and also agricultural drainage to this water table could be the probable reason for this difference.

**Table 7:** Rotated factor loadings of PCA application for heavy metals

**Parameters**	**PC1**	**PC2**	**PC3**
**B**	**0.913**	−0.052	−0.315
**Sn**	**0.881**	−0.037	−0.151
**Hg**	**0.871**	0.007	0.214
**Br**	−0.319	**0.877**	−0.033
**Cr**	−0.103	**−0.859**	0.047
**Ba**	0.521	**−0.635**	0.043
**Zn**	−0.169	**−0.571**	0.023
**Cu**	−0.278	**0.504**	0.411
**As**	−0.099	0.406	**−0.822**
**Al**	−0.274	0.495	**0.613**
**Eigenvalue**	3.790	2.165	1.283
**Variance %**	37.905	21.650	12.835
**Cumulative %**	37.905	59.555	72.390

Numbers in bold indicate effective parameter in each PC.

Application of CA is useful for classifying groundwater in the whole region and makes adequately serving for spatial assessment possible in an optimal manner. Therefore, the number of sampling sites and cost in the monitoring network is reduced without losing any significance of the outcome [28,29].

## Conclusion

This study aimed to examine quality of drinking groundwater of rural communities in Tabriz. The results represented whether the water was suitable or unsuitable for drinking purposes in this area. It was also observed that villages like Eskandar and Olakandi had low quality drinking water. It is suggested to take some necessary measures for supplying desirable water to the people living in these villages.

The dominant cations were in the order of Ca^2+^ > Na^+^ > Mg^2+^ > K^+^ and dominant anions are in the order of HCO_3_^−^ > Cl^−^ > SO_4_^2−^ > NO_3_^2−^. So, hydrochemical faces of water were dominated by Ca HCO_3_.

In this work, different multivariate statistical techniques were used to evaluate variations in groundwater quality. Cluster analysis grouped sampling sites to four clusters of similar water quality characteristics. Based on the obtained data, a future, optimal sampling strategy can be designed which could reduce the number of sampling sites and associated costs. Principle component analysis helped in identifying the factors or sources responsible for variations in water quality.

The results showed arsenic contamination in most of groundwater resources in the western areas. Universally, there are two sources for arsenic contamination in groundwater, which include geogenic source that is sometimes called background or natural and also anthropogenic sources [30,31]. The above-mentioned information can be useful for practical management of arsenic contamination problem and can provide an appropriate perspective for decision making and treatment strategies, especially for point of entry (POE) methods. Undoubtedly, application of different treatment methods that have been well discussed in the literature [32] should be considered an approach after pilot studies using real water samples for arsenic removal. Among the new methods for arsenic removal, iron-amended biosand filters [33] and iron filter [34], electrochemical coagulation [35] and modified granular activated carbon [36,37] could be also considered. Therefore, it is recommended to conduct an extensive study in western areas of Tabriz to find source(s) of arsenic contamination, release mechanisms, distribution and periodic fluctuations of arsenic in the aquifers.

## Competing interests

The authors declare that they have no competing interests.

## Authors’ contributions

The overall implementation of this study including design, experiments and data analysis were done by MM and MP and manuscript was drafted by MP. All authors have made extensive contribution into this study and critically reviewed the article. All authors read and approved the final manuscript.
